# Identification of the potential biological target molecules related to primary open-angle glaucoma

**DOI:** 10.1186/s12886-022-02368-0

**Published:** 2022-04-23

**Authors:** Hongyu Li, Zi Ye, Zhaohui Li

**Affiliations:** 1 Medical School of Chinese PLA, Beijing, China; 2 Senior Department of Ophthalmology, the Third Medical Center of PLA General Hospital, Beijing, China

**Keywords:** Primary open-angle glaucoma, Differentially expressed gene, Competing endogenous RNA network, Bioinformatics analysis

## Abstract

**Background:**

To identify the potential biological target molecules and the corresponding interaction networks in primary open-angle glaucoma (POAG) development.

**Methods:**

The microarray datasets of GSE138125 and GSE27276 concerning lncRNA and mRNA expression profiles in trabecular meshwork of POAG were downloaded from the Gene Expression Omnibus database. The R software was applied to identify differentially expressed (DE) lncRNAs and mRNAs in POAG, and to perform GO and KEGG functional enrichment analysis. Protein–protein interaction (PPI) network and module analysis, and lncRNA-miRNA-mRNA competing endogenous RNA (ceRNA) network were performed by Cytoscape software.

**Results:**

A total of 567 DE-mRNAs were identified from GSE138125 and GSE27276, including 298 up-regulated and 269 down-regulated mRNAs, which were found enriching in biological processes of extracellular matrix organization and epidermis development, respectively. KEGG pathway enrichment analysis further revealed that module genes in PPI network were primarily involved in the AGE-PAGE, PI3K-Akt and TGF-β signaling pathways. Moreover, 897 up-regulated and 1036 down-regulated DE-lncRNAs were identified from GSE138125. Through literature review and databases searching, we obtained 712 lncRNA-miRNA and 337 miRNA-mRNA pairs based on the selected eight POAG-related miRNAs. After excluding 702 lncRNAs and 284 mRNAs that were not comprised in the DE-lncRNA and DE-mRNAs, a total of 53 lncRNA nodes, eight miRNA nodes, 10 mRNA nodes, and 78 edges were included in the final ceRNA network.

**Conclusions:**

This study demonstrated the lncRNA and mRNA expression profiles of trabecular meshwork in POAG patients and the normal controls, and identified potentially ceRNAs and pathways which might improve the pathogenic understanding of this ocular disease.

**Supplementary Information:**

The online version contains supplementary material available at 10.1186/s12886-022-02368-0.

## Background

Glaucoma is currently the second leading cause of blindness in the world, affecting more than 60 million people [1]. According to whether the angle is closed or not, glaucoma can be divided into two categories: closed angle and open angle. Primary open-angle glaucoma (POAG), a common type of glaucoma, is characterized by progressive and irreversible degeneration of retinal ganglion cells and unique visual field loss [2]. It has been determined that a variety of risk factors may be related to the onset of POAG, such as age, elevated intraocular pressure (IOP), family history, etc. [3–5]. In addition, genetics has also been shown to play a key role in the pathogenesis of POAG [6, 7].

A non-coding RNA (ncRNA) is an RNA molecule that is not translated into protein. It has been found that ncRNAs are the main regulators involved in various biological pathological processes [8, 9]. Long non-coding RNA (lncRNA) is a typical ncRNA of more than 200 nucleotides, with fewer exons and tissue- or cell-specific characteristics [10]. LncRNA can regulate gene expression at different biological levels, such as gene translation and transcription [11, 12]. Recently, new evidence has shown that lncRNA is involved in the development of POAG [7, 13]; however, so far, the functions and related mechanisms of most lncRNAs have not been fully elucidated, and only a small portion being well-annotated. In addition, another ncRNA – microRNA (miRNA) has also been proved to play a vital role in the diagnosis and treatment of glaucoma [14–17]. MiRNA is a single-stranded nucleotide with a length of 18–23 bp, which could regulate the expression of target genes at the post-transcriptional level [18]. Salmena et al. put forward a hypothesis that lncRNAs were emerging as competing endogenous RNAs (ceRNAs), communicating with messenger RNA (mRNA) through competitive miRNA [19]. On the one hand, miRNAs can bind to their target mRNAs and inhibit their expression in the ceRNA network. On the other hand, lncRNA can share miRNA response elements (MREs) with mRNA, which can alleviate the inhibition of the miRNA-mediated gene-encoded protein level [20, 21].

Gene Expression Omnibus (GEO; https://www.ncbi.nlm.nih.gov/geo/) is a largest high-throughput database containing microarray- and sequence-based data from a variety of tissues. Mining and analyzing of the vast reliable gene expression data is helpful for revealing molecular changes of diseases [22]. Over the past decades, several studies have utilized the GEO data to identify the underlying molecular mechanisms of glaucoma pathogenesis [7, 23, 24]. Although certain protein-coding genes or miRNAs have been identified, all of them were found in aqueous humor or optic nerve head tissue but not in trabecular meshwork of POAG patients and the interaction network of these genes is seldom reported [25]. In this study, we first obtained differentially expressed RNAs (DE-RNAs), including DE-lncRNAs and DE-mRNAs, in trabecular meshwork of POAG patients by mining two GEO datasets. Subsequently, we performed functional enrichment and protein–protein interaction (PPI) analysis on the acquired DE-mRNA. Then, through the integration of relevant lncRNA, miRNA and mRNA, a regulatory ceRNA network related to POAG was successfully established. This new method of predicting disease-specific ceRNA networks can clarify the regulatory mechanism of lncRNA-mediated ceRNA in the development and prognosis of POAG, and identify new lncRNAs as potential diagnostic biomarkers or therapeutic targets.

## Methods

All data of this study was conducted based on the public databases and performed in accordance with the ethical standards stated in the 2013 Declaration of Helsinki. The informed consent from each participant and the ethical approval was obtained in the original study.

### Data collection and preprocessing

We searched the GEO database for studies comparing RNA expression of human TM between POAG and normal eyes. The RNA expression profile data of GSE27276 based on the GPL2507 platform (Sentrix Human-6 Expression BeadChip), and GSE138125 based on the GPL21827 platform (Agilent-079487 Arraystar Human LncRNA microarray V4), were selected for subsequent analysis. As the lncRNA microarray includes the expression results of lncRNA and mRNA, the lncRNA microarray of GSE138125 is divided into lncRNA array and mRNA array for further analysis. Finally, four pairs of POAG and normal samples in GSE138125 dataset, and 17 POAG samples and 19 normal samples in GSE27276 dataset, were enrolled in this study (Supplementary Tables 1–2). All TM tissues were obtained from POAG patients undertaking the conventional trabeculectomy and the donated normal eyes in both datasets [26].

### Differential expression analysis of RNAs

The probes in gene expression matrix were annotated with corresponding gene symbols based on the platform annotation information. For a given gene mapping to several probes in one sample, the gene expression level was determined as the average of the detected probe values. We used the “limma” package in R to identify the DE-RNAs, and the *p*-value was adjusted to false discovery rate (FDR) by applying the Benjamini–Hochberg (BH) program [27]. FDR adjusted *p*-value < 0.05 and |log_2_Fold Change (FC)|> 0.58 were considered as the cutoff values for DE-RNAs screening. The “ggplot2” and “pheatmap” packages were applied for data visualization via volcano plots and two-way hierarchical clustering heat maps in R [28]. Moreover, the DE-mRNAs from both microarrays were subjected to Venn analysis using the “venn” package in R. Overlapping genes were considered as co-expression DE-mRNAs (Co-DE-mRNAs) in the following analyses.

### Functional enrichment analysis

To understand the potential biological functions of DE-mRNAs, the “clusterProfiler” package in R was utilized to identify the Gene Ontology (GO) biological functions and Kyoto Encyclopedia of Genes and Genomes (KEGG) pathways [29, 30]. The GO and KEGG analyses were performed for up-regulated and down-regulated DE-mRNAs, separately. Records with a *p*-value < 0.05 and enrichment > 2.0 were regarded as statistically significant in functional enrichment analysis. The “enrichplot” package in R was utilized to show the relationship between GO-terms and genes by chord plots.

### Construction of PPI network

Search tool for the retrieval of interacting genes (STRING, http://string-db.org/), an online database that collects predicted and/or testified protein interactions, was used to construct the PPI interaction network of the identified DE-mRNAs [31]. After DE-mRNAs were entered into the database, important PPI interaction pairs were obtained from six sources: neighborhood, gene fusion, co-occurrence, co-expression, experiments, databases, and text mining. Cytoscape software was used to predict the association among these target genes with a combined confidence score ≥ 0.4 in the regulatory network analysis. In addition, the MCODE program in Cytoscape was used to identify important modules (degree cutoff ≥ 2, node score cutoff ≥ 0.2, K core ≥ 2 and maximum depth = 100). The clusters that contained ≥ 10 nodes and that possessed MCODE scores ≥ 10 were selected [32].

### Identification and establishment of potential ceRNA interactions

**Fig. 1:**
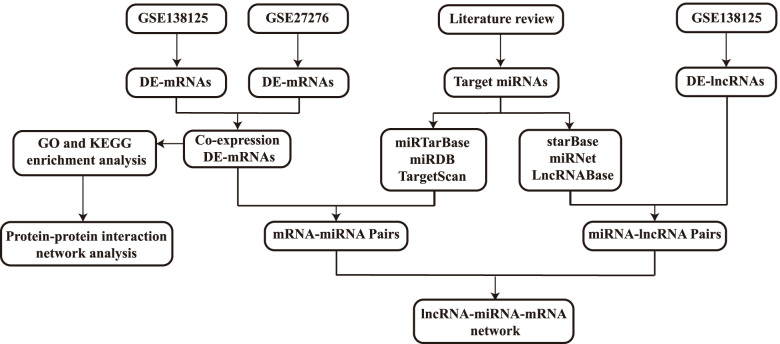
The flowchart of the identification differentially RNAs and the subsequent construction of lncRNA-miRNA-mRNA network. DE-RNAs, differentially expressed mRNAs, DE-lncRNAs, differentially expressed lncRNAs

Based on the hypothesis that lncRNA can be anchored to MREs to prevent the binding of miRNA to its target mRNA, a ceRNA network was constructed [33]. First, we selected several miRNAs closely related to the pathogenesis of POAG through literature review. These miRNAs directly promote the development of POAG by participating in pathological processes such as TM extracellular matrix deposition or inflammation [17]. The miRNA-lncRNA interactions were predicted by the miRNet [34, 35] (https://www.mirnet.ca/), LncRNABase (https://starbase.sysu.edu.cn/starbase2/mirLncRNA.php) and starBase [36, 37] (https://starbase.sysu.edu.cn/). The miRDB (http://www.mirdb.org/), miRTarBase [38] (http://mirtarbase.mbc.nctu.edu.tw/) and TargetScan (http://www.targetscan.org/) were used for predicting target mRNAs of miRNA. The lncRNAs and mRNAs, existing in the DE-lncRNA and DE-mRNA groups, respectively, and also appearing in the above databases, were enrolled in the final ceRNA network (Fig. 1). Cytoscape software was used for the construction and visualization of the lncRNA-miRNA-mRNA ceRNA network [39].

## Results

### Screening of significantly DE-RNAs in POAG

**Fig. 2:**
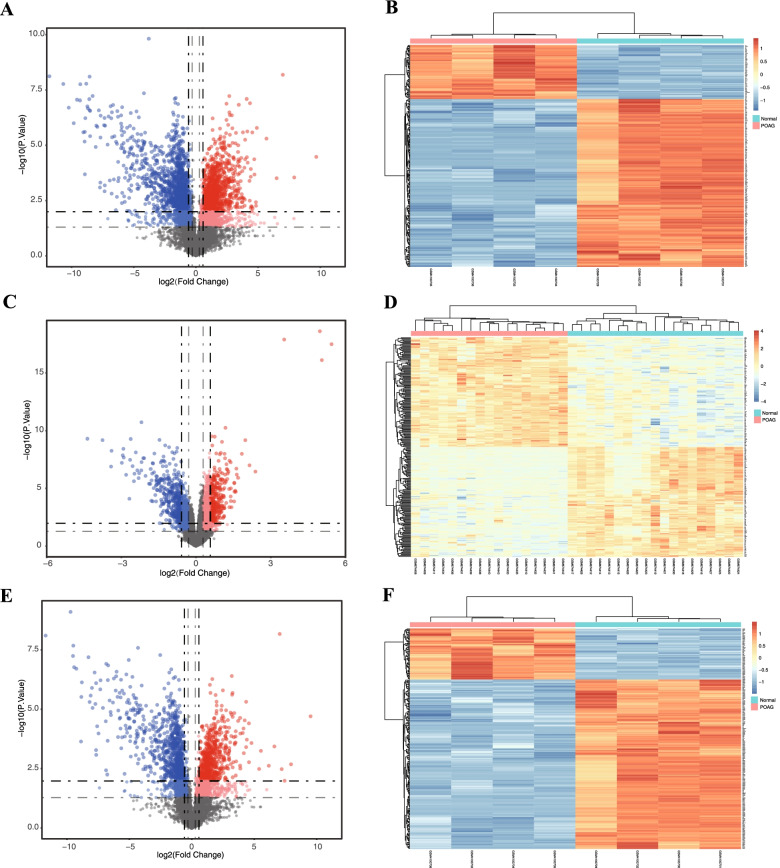
Overview of differentially expressed RNAs from microarray datasets. Volcano plots and heat maps of the differentially expressed mRNAs from GSE138125 **A, B** and GSE27276 **C, D**, and differentially expressed lncRNAs from GSE138125 **E, F**. Dash-dotted lines: vertical ones represent log transformed *p* value, and horizontal ones indicated the mean expression differences of genes between POAG and normal samples. Red dots are for up-regulated RNAs and blue dots for down-regulated ones. Color scale bar denotes the expression levels from high (dark red) to low (dark blue). |log_2_FC|> 0.58 and adj. *p*-value < 0.05 were set as the cut-off criteria

**Fig. 3:**
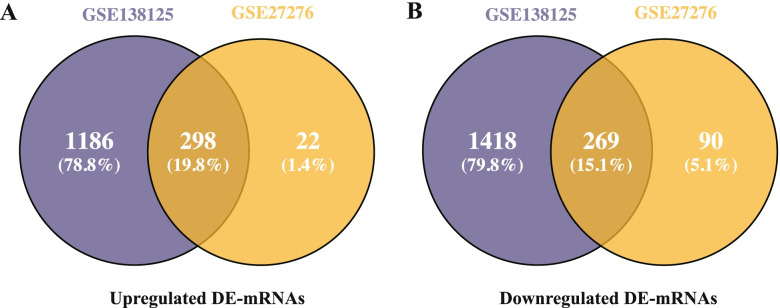
Venn diagrams of overlapped differentially expressed mRNAs (DE-mRNAs) in GSE138125 and GSE27276

All microarray data is standardized after median normalization. The volcano plots and heat maps of both DE-mRNAs and DE-lncRNAs are shown in Fig. 2, showing that the DE-RNAs in both datasets could be easily distinguished from each of the samples, and suggesting an expressional heterogeneity among the samples. A total of 567 DE-mRNAs were identified after screening of GSE138125 and GSE27276, among which 298 were up-regulated and 269 were down-regulated (Fig. 3). Moreover, 897 up-regulated and 1036 down-regulated DE-lncRNAs were identified after screening of GSE138125. Table 1 shows the top 10 up- and down-regulated DE-mRNAs from GSE138125 and GSE27276, and top 10 up- and down-regulated DE-lncRNAs from GSE138125.

**Table 1:** The top 10 up- and down-regulated DE-mRNAs and DE-lncRNAs

mRNA	Log_2_FC	*p* Value	lncRNA	Log_2_FC	*p* Value	Change
HBA2	4.65	3.42E-11	**AC068282.1**	9.51	1.92E-05	UP
HBB	4.51	9.67E-14	**AC105916.1**	7.95	2.02E-03	UP
HBD	2.89	3.90E-08	**AC022517.1**	7.43	9.72E-03	UP
MGP	2.08	5.19E-07	**LINC02428**	7.20	3.22E-03	UP
HLA-DPA1	1.74	1.87E-08	**NTN5**	7.04	6.55E-09	UP
ECRG4	1.60	2.28E-07	**DPP9-AS1**	6.61	3.58E-04	UP
PTGDS	1.54	3.18E-08	**LINC02682**	6.16	2.32E-03	UP
CYTL1	1.51	4.24E-06	**AC079790.2**	6.00	2.32E-02	UP
GRP	1.48	1.16E-05	**AC096577.1**	5.53	5.49E-04	UP
HBG1	1.43	4.89E-06	**LINC02489**	5.39	3.17E-03	UP
KRT13	-2.69	4.95E-07	**AL138787.2**	-11.67	7.66E-09	DOWN
KRT19	-2.68	4.26E-04	**AC006305.1**	-9.68	7.69E-10	DOWN
LCN2	-2.30	3.00E-06	**AC136443.4**	-9.49	5.53E-08	DOWN
S100A9	-2.27	6.62E-06	**POM121L4P**	-9.46	2.03E-08	DOWN
SLPI	-2.27	1.37E-06	**AC002454.1**	-9.40	1.65E-07	DOWN
PAX6	-2.24	4.12E-06	**AL358876.2**	-9.22	1.90E-07	DOWN
S100A8	-2.10	3.75E-04	**AC008758.6**	-8.85	2.22E-04	DOWN
TNNT3	-2.08	3.67E-08	**PDE6B**	-8.82	2.94E-06	DOWN
TGM1	-1.97	5.53E-06	**PARD6G-AS1**	-8.78	7.15E-07	DOWN
KRT5	-1.86	4.17E-05	**LINC02008**	-8.25	6.24E-08	DOWN

### Functional enrichment analysis

**Fig. 4:**
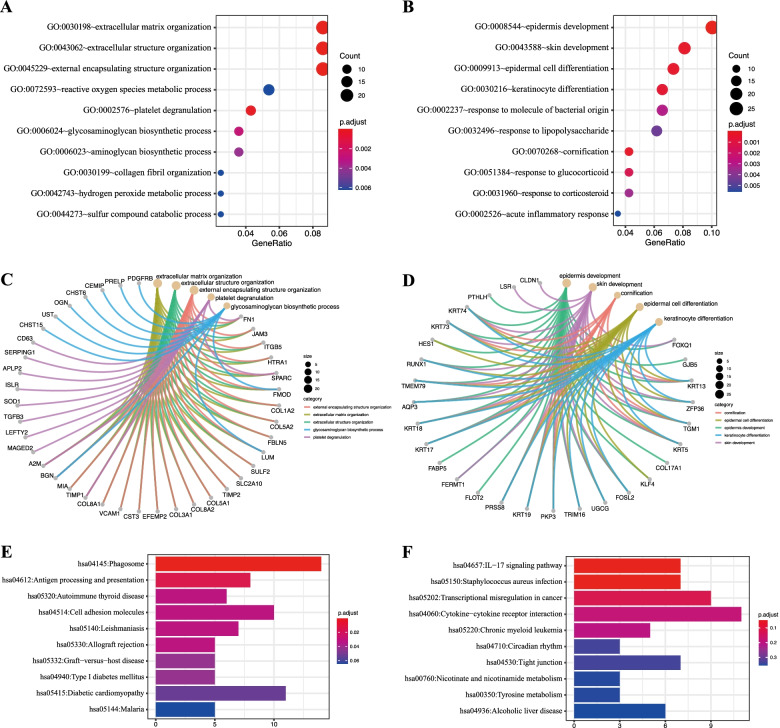
Functional enrichment analysis of the 567 overlapped differentially expressed mRNAs (DE-mRNAs). **A** and **B**, The top 10 enriched biological processes for the up-regulated and down-regulated DE-mRNAs via GO analysis; **C** and **D**, Chord plots revealed the detailed relationships between GO-terms and up-regulated and down-regulated DE-mRNAs; **E** and **F**, The top 10 enriched KEGG pathways for the up-regulated and down-regulated DE-mRNAs. (KEGG pathway database is available at www.kegg.jp/feedback/copyright.html)

The complete results of GO analysis, including biological process (BP), cellular component (CC) and molecular function (MF), and KEGG analysis are presented in Supplementary Table 3. The top 10 GO-BP terms of DE-mRNAs were visualized in the bubble diagram (Fig. 4A-B). The top three enriched GO term of up-regulated DE-mRNAs were the extracellular matrix organization (GO:0,030,198), extracellular structure organization (GO:0,043,062) and external encapsulating structure organization (GO:0,045,229). The epidermis development (GO:0,008,544), skin development (GO:0,043,588) and epidermal cell differentiation (GO:0,009,913) were the top three enriched pathways of down-regulated DE-mRNAs. GO chord plot shows the relationship between GO term and genes (Fig. 4C-D). In addition, the top 10 KEGG pathways enriched are visualized in the bar plots (Fig. 4E-F), which shows that immunity and infection probably played an important role in POAG.

### PPI network analysis

**Fig. 5:**
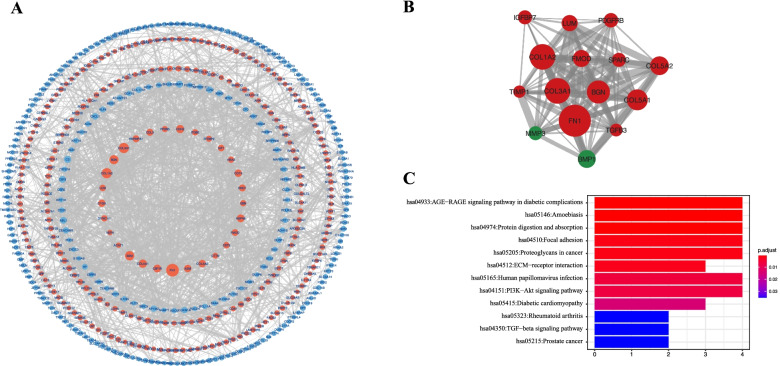
Protein–protein interaction analysis. **A**, Protein–protein interaction network of the differentially expressed mRNAs from GSE138125 and GSE27276 datasets; **B,** The sub-network module genes analyzed by MCODE in Cytoscape software; **C, **KEEG pathways analysis of the genes in the sub-network module genes

PPI network of the significantly DE-mRNAs was constructed with 133 nodes and 259 edges being mapped from STRING database (Fig. 5A). Only one cluster was selected from the PPI network by MCODE analysis based on the criterial of nodes ≥ 10 and scores ≥ 10 (Fig. 5B). The cluster consisted of eight up-regulated genes (FN1, COL1A2, COL3A1, BGN, COL5A1, COL5A2, FMOD, LUM, PDGFRB, SPARC, IGFBP7, TGFB3, TIMP1) and two down-regulated genes (BMP1, MMP3). KEGG pathway enrichment analysis revealed that these module genes were primarily involved in the AGE-PAGE, PI3K-Akt and TGF- β signaling pathway (Fig. 5C).

### Construction of a ceRNA network

**Fig. 6:**
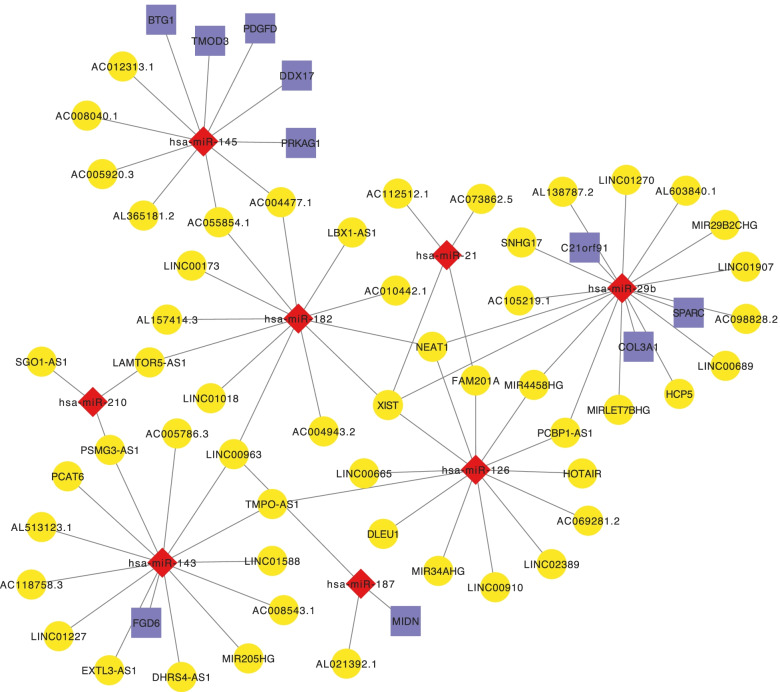
Competing endogenous RNA network in human POAG. Red nodes denote miRNAs, blue for mRNAs and yellow for lncRNAs

To further understand how lncRNA regulates mRNA through binding with miRNA in human POAG, a lncRNA-miRNA-mRNA (ceRNA) network was constructed. By literature review, we first identified eight miRNAs that may be related to the pathogenesis of POAG, including hsa-miR-29b, hsa-miR-143/145, hsa-miR-21, hsa-miR-210, hsa-miR-126, hsa-miR-182 and hsa-miR-187. Then we found 712 lncRNAs interacting with the eight miRNAs via searching the miRNet, LncRNABase and starBase databases. However, only 53 of these lncRNAs were overlapped with the DE-lncRNAs of GSE138125. Similarly, through searching miRDB, miRTarBase and TargetScan databases, we found 10 mRNAs interacting with the target miRNAs as well as contained in the Co-DE-mRNAs between GSE138125 and GSE27276. As shown in Fig. 6, the preliminarily lncRNA-miRNA-mRNA network was built based on the miRNA-mRNA and miRNA-lncRNA pairs, which was composed of 53 lncRNA nodes, eight miRNA nodes, 10 mRNA nodes, and 78 edges.

## Discussion

POAG is a progressive optic neuropathy, and it is estimated that by 2040, there will be 111.8 million glaucoma patients worldwide [40]. The major risk factor of POAG is the increased IOP, which could compress the structures in and around the optic nerve head to disturb the axoplasmic transport of nerve fibers [41]. This can lead to the death of retinal ganglion cells and their axons, resulting in thinning of the edge of the neural retina and depression of the optic nerve head [42]. Injury related to elevated IOP is mainly manifested as the occurrence of TM degeneration [43]. TM is a key component of the aqueous humor outflow pathway and constitutes most of the outflow resistance [44]. In POAG, a series of pathological changes occur in the TM, leading to increased outflow resistance and elevated IOP [45]. In the past few years, considerable efforts have been made to explore the molecular mechanisms of POAG [46, 47]. However, most studies have focused on protein-coding genes or miRNAs [15, 48]. Neither GO or KEGG analysis of key genes nor the lncRNA expression profile of the TM in POAG was established. Therefore, we analyzed two published microarray data from GEO databases, including the lncRNA microarray from GSE138125 and mRNA microarray from GSE138125 and GSE27276, and constructed a possible ceRNA network based on the DE-mRNAs and DE-lncRNAs. To our knowledge, this study is the largest comparison of lncRNA and mRNA expressions in the TM of POAG group and normal group.

In this study, a total of 567 significantly DE-mRNAs were found in POAG patients compared to normal beings, including 298 up-regulated and 269 down-regulated DE-mRNAs. Functional analysis further showed that the main enrichment pathways of the up-regulated DE-mRNAs resided in cell organization, such as extracellular matrix organization involving genes of fibromodulin (FMOD, log_2_FC = 1.40), biglycan (BGN, log_2_FC = 1.25) and HtrA serine peptidase 1 (HTRA1, log_2_FC = 1.16), etc. Specifically, HTRA1 and FMOD are responsible for the degradation and reconstruction of the extracellular matrix [49], while BGN is positively correlated with collagen fibril assembly in multiple tissues [50, 51]. Previous study indicated that both FMOD and BGN play a role in the pathogenesis of POAG, in which BGN participate in the extracellular matrix remodeling and axonal damage in the lamina cribrosa of the optic nerve head in glaucomatous optic neuropathy, and FMOD might be associated with susceptibility to glaucoma damage [52–54]. In addition, it was reported that HTRA1 participate in the extracellular deposits of proteins and lipids on the basal side of retinal pigment epithelium, which contribute to the pathogenesis of age-related macular degeneration [55]. For the down-regulated DE-mRNAs, development related pathways were mainly enriched. The pathway of epidermis development, for example, involved genes of keratin 13 (KRT13, log_2_FC = -2.69), keratin 19 (KRT19, log_2_FC = -2.68) and transglutaminase 1 (TGM1, log_2_FC = -1.97), etc. Both KRT13 and KRT19 are members of the keratin family which is a group of intermediate filament proteins responsible for the structural integrity of epithelial cells. And TGM1 is also involved in the process of keratinization. The above three mRNAs mainly exist in epithelial cells, which may be involved in the process of corneal epithelial conjunctivalisation, and few studies have investigated their relationship with POAG [56]. These down-regulated DE-mRNAs may be considered as potential targets for drug therapy of POAG. Moreover, the PPI network of DE-mRNAs outlined their functional connections and revealed a total of 15 hub genes: FN1, COL1A2, COL3A1, BGN, COL5A1, COL5A2, FMOD, LUM, PDGFRB, SPARC, IGFBP7, TGFB3 and TIMP1 amongst the up-regulated genes, and BMP1, MMP3 amongst the down-regulated genes. Among them, the top DE-mRNA was fibronectin (FN1), a glycoprotein within the extracellular matrix. It has been proved that FN1 was upregulated in tears, tenon's capsule and aqueous humor samples in pseudoexfoliation glaucoma, and that inhibiting FN1 would promote the proliferation and invasion of TM cells [57, 58]. Two other highly expressed mRNAs, COL1A2 and COL3A1, have also been shown to be genetic and biochemical biomarkers of POAG [59, 60]. All above significantly dysregulated genes involved in extracellular matrix organization and epidermis development may play a vital role in the pathogenesis of POAG and thus deserve more exploration.

Moreover, 897 up-regulated and 1036 down-regulated DE-lncRNAs were identified by screening the GSE138125 database. Compared with protein-coding gene and miRNA, lncRNA has significant advantages as a prognostic biomarker or therapeutic target [61, 62]. LncRNA regulates the level of gene-encoded proteins by competitively binding to MREs to regulate cell activities [63]. For example, the lncRNA TGFβ2-AS1 could promote the production of extracellular matrix production through targeting TGF-β2 in human TM cells, suggesting that lncRNA TGFβ2-AS1 may be a potential treatment target for POAG [64]. Moreover, lncRNAs are also important components of the ceRNA network, which plays an important role in the post-transcriptional regulation of genes. It has reported that the ceRNA was associated with the molecular mechanisms of eye disease [65]. In these circumstances, a ceRNA network based on the differentially expressed genes in TM tissue can be helpful to understand the underlying molecular mechanisms of POAG development. In order to construct the lncRNA-miRNA-mRNA network, we selected a total of eight miRNAs that have been functionally proved in cells and animal models to be associated with the pathogenesis of POAG, including hsa-miR-29b, hsa-miR-143/145, hsa-miR-21, hsa-miR-210, hsa-miR-126, hsa-miR-182 and hsa-miR-187. All these miRNAs are involved in IOP elevation or TM damage. For example, the hsa-miR-29b could inhibit the expression of collagen I/III/IV through PI3K/Akt/Sp1 signaling pathway, leading to the deposition of extracellular matrix in the TM [66]. And the hsa-miR-143/145 primarily promotes the phosphorylation of myosin in TM cells, which in turn promotes the contraction of TM cells and thus results in an elevated IOP [67, 68].

In this study, all above eight miRNAs were applied to predict the possible miRNA-lncRNA pairs and miRNA-mRNA pairs within the public databases. These identified lncRNAs or mRNAs presented both in the pairs and in the DE-lncRNAs or DE-mRNAs groups were enrolled in the final ceRNA network. Consequently, 53 lncRNAs, eight miRNAs, and 10 mRNAs were selected for the ceRNA network analysis. Among them, the most up-regulated lncRNA in the ceRNA network is HOTAIR, which is an antisense lncRNA that has been reported to play a critical role in multiple complex diseases, such as the progression of Parkinson’s disease, psoriasis and cancers, by targeting hsa-miR-126 and its downstream pathways [69–71]. In POAG patients, hsa-miR-126 may be down-regulated under chronic hypoxia conditions, resulting in retinal ganglion cells injury through targeting VEGF-Notch signaling pathway [72]. We speculated that this can be related to the competitive binding of up-regulated HOTAIR to the hsa-miR-126 response element. Besides, the ceRNA network also shown that LINC00173 and LBX1-AS1 could sponge to the hsa-miR-182. Recent studies suggested that hsa-miR-182 was up-regulated in aging TM cells and aqueous humor of POAG patients to regulate IOP and protect retinal ganglion cells from oxidative stress [73, 74]. Thus, we thought the elevated IOP and optic nerve damage of POAG would be associated with the up-regulation of LINC00173 and LBX1-AS1. XIST is another notable lncRNA in the ceRNA network, which is interacted with four miRNAs including hsa-miR-29b, hsa-miR-21, hsa-miR-126 and hsa-miR-182. It has been reported that XIST was involved in the epithelial-mesenchymal transitions of retinoblastoma and also participated in the apoptosis and migration of retinal pigment epithelial cells subjected to hyperglycemia [75, 76]. However, few studies have reported the function of XIST as miRNA sponges in the development of POAG.

## Conclusions

In summary, current research demonstrated the lncRNA and mRNA differential expression profiles of TM between POAG patients and the normal controls by bioinformatics methods. We identified several potential lncRNAs and mRNAs that may be involved in the pathogenesis of POAG. And the lncRNA-miRNA-mRNA ceRNA network is successfully constructed, showing complex interactions among the lncRNAs, miRNAs and mRNAs during the development of POAG. This may help reveal the unknown pathogenesis and potential therapeutic targets of POAG. Future research effort should focus more on lncRNA exploration to clarify the molecular mechanisms concerning the pathogenesis of POAG.

## Supplementary Information

**Additional file1:** **Supplementary Table 1.** Clinical data of the enrolled patients in GSE27276 dataset. **Supplementary Table 2.** Clinical data of the enrolled patients in GSE138125 dataset. **Supplementary Table 3.** Enriched functional annnotation terms associated with significantly up-regulated genes in primary open-angle glaucoma.   

## Data Availability

The datasets supporting the conclusions of this study are available in the Gene Expression Omnibus (GEO, https://www.ncbi.nlm.nih.gov/geo/). (GSE138125: https://www.ncbi.nlm.nih.gov/geo/query/acc.cgi?acc=GSE138125; GSE27276: https://www.ncbi.nlm.nih.gov/geo/query/acc.cgi?acc=GSE27276).

## Abbreviations

BH: Benjamini–Hochberg
BP: Biological process
CC: Cellular component
CeRNA: Competing endogenous RNA
Co-DE: Co-expression differentially expressed
DE: Differentially expressed
FC: Fold change
FDR: False discovery rate
GEO: Gene Expression Omnibus
GO: Gene Ontology
IOP: Intraocular lens pressure
KEGG: Kyoto Encyclopedia of Genes and Genomes
LncRNA: Long non-coding RNA
MF: Molecular function
MiRNA: MicroRNA
MRE: MicroRNA response element
MRNA: Messenger RNA
POAG: Primary open-angle glaucoma
PPI: Protein-protein interaction
TM: Trabecular meshwork
